# Validation in diabetic rats of a fully automated insulin delivery system based on impulsive offset-free MPC control

**DOI:** 10.1371/journal.pone.0330121

**Published:** 2025-09-04

**Authors:** Jhon E. Goez-Mora, Natalia Arbelaez-Córdoba, Norman Balcazar-Morales, Pablo S. Rivadeneira

**Affiliations:** 1 Grupo GITA, Facultad de Minas, Universidad Nacional de Colombia, Medellín, Colombia; 2 Grupo GENMOL, Departamento de Fisiología y Bioquímica, Facultad de Medicina, Universidad de Antioquia, Medellín, Colombia; University of Oxford, UNITED KINGDOM OF GREAT BRITAIN AND NORTHERN IRELAND

## Abstract

The development of an impulsive automated insulin delivery system (i-AiDS) for type 1 diabetes mellitus aims to provide real-time blood glucose regulation with minimal human intervention. This study presents the validation of an offset-free impulsive zone model predictive control strategy designed to cope with external disturbances such as meal intake and plant-model mismatch in a diabetic rat model. Fourteen male Wistar rats induced diabetes with streptozotocin were monitored using an continuous glucose monitoring and regulated by delivering insulin with a customized low-cost pump. After acquiring diabetes condition, the procedure for installing the devices in the rat is carried out. During the first day, manual insulin injections are made by the pump, the glucose response is recorded by the interface and an off-line parametric estimation is executed. Based on the parameters found, simulations are used for the first tuning of the controller and the estimator. During the second day, the parameters of the model and the control are tested and adjusted. Finally, on the third day, a 72-hour test of the impulsive begins in full autonomous mode. Results showed that the controller achieved an average of 83.4% of the time within the target range of 80-180 mg/dL, with no severe hypoglycemic or hyperglycemic events. The median absolute relative difference between model predictions and actual sensor data was 24.66%, indicating the presence of plant-model mismatch that was effectively handled by the controller. Peak hyperglycemic events reached 320 mg/dL, but were regulated within 50 minutes, while mild hypoglycemic events occurred in 3.62 ± 1.8 cases per subject. The study demonstrates the efficacy of the controller in managing unannounced carbohydrate intake and physiological disturbances in a real-world preclinical environment. These findings provide a foundation for future clinical trials, emphasizing the importance of in vivo validation of control strategies to refine the i-AiDS for human use. Improvements in model accuracy and dynamic parameter tuning could further enhance performance, particularly in longer experimental periods.

## Introduction

Developing an automated insulin delivery system (AiDS) has become one of the leading research studies focused on treating type 1 diabetes mellitus (T1DM) nowadays. Still, in development, several technical and ethical challenges remain to be resolved. One of the most significant hurdles is the complexity of modeling the glucose-insulin dynamics, which can be hard to predict and manage in real-time due to the large number of variables that can affect such dynamics in the process. Even when combined with continuous glucose monitors (CGM), insulin delivery systems still require periodic adjustments or human intervention, making it challenging to achieve optimal glycemic control. A fully automated closed-loop system, which can monitor blood glucose (BG) levels and adjust insulin delivery without manual input, is the goal of AiDS research. However, this brings forth control strategies, algorithm accuracy, and safety challenges [1–6].

Model Predictive Control (MPC) is one of the most promising control strategies being explored in this problem. This controller strategy has emerged as a leading candidate for AiDS prototypes due to its capabilities [7], which can be obtained through an optimization process and the possibility of integrating constraints such as limits on the minimum and maximum insulin dosage, the maximum and minimum glucose ranges or states in the model, the amount of insulin on board [8], or even variables related to the amounts of insulin available in the reservoir or energy consumption. MPC utilizes mathematical models of glucose-insulin interactions to forecast how current states will evolve over a prediction horizon. With its structure for calculating insulin dosage, MPC can handle disturbances such as unannounced meals, varying physical activity levels, and the inherent delays in insulin absorption and action. This approach makes the MPC present favorable characteristics for handling BG complex and its nonlinear nature in T1DM.

In the last decade, several variations of MPC have been proposed to address specific challenges in AiDS development. One approach involves an adaptive personalized MPC, which adjusts control parameters based on recent data from the patient’s glucose measurements and insulin delivery history. This allows the system to dynamically modulate the control strength depending on the individual’s metabolic needs. Another promising enhancement is the inclusion of an automatic Bolus Priming System (BPS)[9], administering additional insulin doses when detecting unannounced meal intake. Simulation studies have demonstrated the efficacy of these advanced MPC strategies in improving time-in-range and reducing events of hyperglycemia.

Nonlinear MPC formulations have also been explored to handle the more complex dynamics observed in T1DM. For example, impulsive control models where insulin is delivered as discrete impulses rather than continuous infusions have shown promise in improving glucose regulation, particularly in cases where insulin sensitivity varies over time [10, 11]. This nonlinear approach has been successfully applied to both glucose regulation and other biomedical applications, such as oncolytic virus therapy, demonstrating its versatility [12].

Another advancement in MPC design is the offset-free control approach, which compensates for plant-model mismatches, a common issue in biological systems due to physiological variations. Offset-free zone MPC (OF-ZMPC) strategies incorporate disturbance estimation techniques to correct the deviation between the model’s predictions and the actual glucose levels, ensuring more accurate insulin dosing even in the presence of changes in the patient’s metabolism [13]. These methods have been tested in virtual environments, using simulations of patients with T1DM in different age ranges and parametric variations over 30% of the mathematical model, where they have shown significant improvements in maintaining normoglycemia and reducing the incidence of hypoglycemia, even in scenarios with unannounced meals and sensor noise [14, 15].

To summarize, impulsive systems offer a robust framework for modeling systems where changes can occur abruptly. In diabetes, impulsive events correspond to an insulin bolus or a sudden rise in glucose after meals. By considering an impulsive model, the controller can respond and accurately predict the nature of glucose dynamics. In parallel, the MPC has gained prominence for its ability to anticipate future glucose levels and adjust insulin doses accordingly. However, traditional MPC can exhibit steady-state errors when faced with model mismatch. To address this, the offset-free ZMPC incorporates mechanisms that ensure the controller can drive blood glucose levels to a target range without residual error, even in the presence of unmodeled influences. This is especially valuable in clinical settings where physiological variability and unannounced events are common. Together, OF-ZMPC and impulsive system modeling enable more reliable and physiologically realistic control, contributing to the development of safer and more effective impulsive automated insulin delivery systems (i-AiDS) therapies.

Despite the significant potential of MPC in i-AiDS development a major hurdle remains the validation of these control strategies before they can be tested in human clinical trials. Developing an i-AiDS requires rigorous testing, traditionally involving animal trials. Animal models, such as diabetic rats, have been essential in understanding the physiological dynamics of glucose-insulin interactions. These preclinical trials allow researchers to test the safety and efficacy of different control algorithms and insulin delivery systems in a controlled environment [16–19]. However, there are ethical concerns and limitations associated with the use of animals in research, including variability in how these models translate to human physiology and the welfare of the animals involved.

In-silico simulations have been used as a powerful alternative for preliminary testing to address these concerns. These simulations use mathematical models and computer algorithms to mimic the glucose-insulin dynamic, allowing researchers to test control strategies without involving animals. While this method can reduce the need for animal trials, it does not entirely eliminate them because even though different simulation scenarios can be configured, it is not possible to consider all the different aspects, variables, or disturbances that the i-AiDS will be affected by during a real test. Simulations must be validated through in vivo testing before they can be applied to human clinical trials. Therefore, a balance between simulation-based and animal-based preclinical testing remains crucial in the i-AiDS development process. Integrating preclinical animal trials with advanced in silico models offers a more ethical and potentially more efficient path to developing safer and more effective i-AiDS solutions [20]. By refining algorithms and control strategies in a simulated environment, researchers can minimize the number of animal tests needed, focusing on validation rather than exploratory trials. This approach not only addresses the ethical concerns of animal testing but also accelerates the development process, bringing the i-AiDS prototype closer to clinical application.

For the development of the experiment in this work, after performing the procedure for the rats to acquire the diabetes condition, a bolus of insulin is injected, and the CGM data is stored to estimate parameters offline. Based on the estimated parameters, a tuning of the controller and the estimator is performed, which is subsequently implemented in the i-AiDS in a test of 72 consecutive hours; the food is left at will and is not announced to the controller. The results show that the proposed controller maintains, on average, 83.4% of the time in the 80-180 mg/dL range, avoiding severe cases of hypo and hyperglycemia. The median absolute relative difference between model predictions and actual sensor data was 24.66%, indicating the presence of plant-model mismatch that the controller effectively handled. i-AiDS BG regulation prevents excessive weight loss; on average, the rats lost 4.3% of body weight by the end of the experiment. The study demonstrates the efficacy of the OF-ZMPC controller in managing unannounced carbohydrate intake and physiological disturbances in a real-world preclinical environment.

This work focuses on performing preclinical trials using animals to test the i-AiDS prototype that implements an OF-ZMPC controller, validating the advantages of incorporating the plant-model mismatch in calculating insulin dosage before performing clinical trials with humans. The first section shows the T1DM model, the structure of the MPC controller, the devices as CGM, and the custom pump used in the test and describes the scenario of the trials, including the animals used, times of trial, procedures, measurements, limitations, and data analysis; in the results, the estimated parameters of the animals, the controller strategy performance and the condition of the animals during the test are reported; in the last section, the results of the preclinical test are discussed, identifying the advantages and limitations of the controller, showing the perspectives and conclusions.

## Materials and methods

### Devices

The Freestyle Libre sensor from Abbott Diabetes Care (version 1) is a disposable sensor with a maximum lifespan of 14 days. The unit reads equivalent glucose values in plasma or interstitial glycemia (IG) concentration. RAW data are accessed via a near-field communication protocol. The transmitter used is Miaomiao version 2, designed to read the sensor via near-field communication and transmit data via Bluetooth. It collects glucose information and can track it every 5 minutes.

For the monitoring and control interface computer, a workstation with a Windows 10 64-bit operating system, 64GB RAM, Intel(R) Xeon(R) W-10855M CPU @ 2.80GHz 2.81 GHz processor, and Qualcomm QCA61x4A Bluetooth is used.

The custom insulin pump is based on the concept of an ultra-low-cost prototype. The baseplate is the low-cost FireBeetle Board ESP32 [V4.0], which integrates an ESP WROOM 32 Wifi & Bluetooth Dual-Core MCU Module. The motor controller is the Texas Instruments DRV8833 H-bridge motor drive. The DC motor is coupled with a worm screw. Hall effect sensors are used for position sensing, collecting 4 data points per revolution. The positioning closed-loop controller is a standard proportional-integral-derivative controller [21].

**Fig 1:**
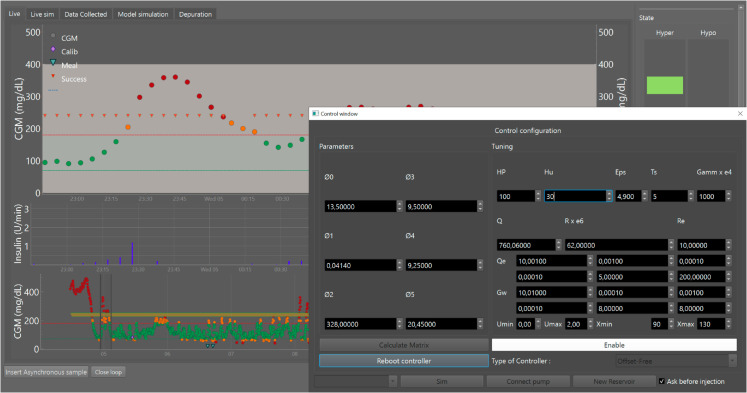
Monitoring and control interface of artificial pancreas system. The central panel contains information on glycemic values using CGM and insulin injected by the pump. The control window includes the configuration of the model parameters, the controller type, the control and estimator tuning, and the maximum and minimum limits of the *x*_1_ state and the insulin units *u*.

Also implemented is a computer-based monitoring interface, previously tested to store and manage glucose data from the sensor and injected insulin [22]. The interface handles all the pump functions, model parameters, tuning controller, and sensor signals shown in Fig 1.

### Controller

#### System dynamics for glucose control.

BG dynamics has typically been represented by mathematical models falling into either maximal or minimal-like models [23]. The model used here is a minimal physiological model based on five compartments representing glucose dynamics, insulin absorption and action, and meal absorption dynamics which guarantees global structural identifiability [24]. The state-space representation of the model is given by

$$\dot{x}(t)=Ax(t)+B_{u}u(t)+B_{r}r(t)+E$$ (1)

with


$$A=\left[\begin{array}{ccccc} -p_{1} & -p_{2} & 0 & p_{3} & 0\\ 0 & -1/p_{4} & 1/p_{4} & 0 & 0\\ 0 & 0 & -1/p_{4} & 0 & 0\\ 0 & 0 & 0 & -1/p_{5} & 1/p_{5}\\ 0 & 0 & 0 & 0 & -1/p_{5} \end{array}\right],$$

$$B_{u}={\left[\begin{array}{c} 0\\ 0\\ \frac{1}{p_{4}}\\ 0\\ 0 \end{array}\right]}^{\prime },B_{r}={\left[\begin{array}{c} 0\\ 0\\ 0\\ 0\\ \frac{1}{p_{5}} \end{array}\right]}^{\prime },E={\left[\begin{array}{c} p_{0}\\ 0\\ 0\\ 0\\ 0 \end{array}\right]}^{\prime }.$$

The measured variable corresponds to the BG concentration, i.e., y=Cx, with $C=\left[\begin{array}{ccccc} 1 & 0 & 0 & 0 & 0 \end{array}\right]$. All model variables and parameters have physiological interpretation, as seen in Table 1.

**Table 1:** Description of variables and parameters of the model.

Variable	Description	Units
*x*_1_	glycemia	mg/dL
*x*_2_,$\;x_{3}$	delivery rates of insulin in the blood and subcutaneous space compartments, respectively	U/min
*x*_4_, *x*_5_	delivery rates of carbohydrates in the stomach and gut, respectively	g/min
*u*	Exogenous insulin	U/min
*r*	Carbohydrates intake	g/min
Parameter	Description	Units
*p*_0_	Endogenous glucose production at zero-insulin level.	mg/dL/min
*p*_1_	Hepatic autoregulation.	1/min
*p*_2_	Insulin sensitivity.	mg/dL/U
*p*_3_	Carbohydrate bioavailability.	mg/dL/g
*p*_4_	Time-to-maximum of effective insulin concentration.	min
*p*_5_	Time-to-maximum appearance rate of glucose.	min

This i-AiDS design has been approached leveraging the concept of impulsive systems [13, 14, 25]. This consideration is appropriate since insulin doses are administered as small-spaced pulses rather than continuous or discrete doses. Adhering to that framework, model (1) can be expanded as an underlying discrete-time subsystem to represent the state’s free response $\left(x^{•}\right)$ and the state after the impulsive input $\left(x^{\circ }\right)$:

$$x^{•}(k+1)=A^{•}x^{•}(k)+B_{u}^{•}u^{•}(k)+B_{r}^{•}r^{•}(k)+E^{•},$$ (2)

where $A^{•}=e^{AT}$, $B_{u}^{•}=e^{AT}B_{u}$, $B_{r}^{•}=\int_{0}^{T}e^{As}dsB_{r}$, $E^{•}=\int_{0}^{T}e^{As}dsE$, and *T* is a constant time of impulses. Note that the input is applied to $x^{•}(k)$, the state directly related to the measured variable. In this regard, model (2) is used in the control system formulation.

#### Offset free model predictive control.

When the prediction model utilized in the control strategy is not accurate enough to describe the plant behavior, i.e., when there is a plant-model mismatch, the MPC can generate a misleading control action that does not steer the system to the target and typically generates a steady state offset.

This section aims to describe the control strategy that drives the system to a nonempty target set by means of pulse control actions while satisfying constraints and compensating for the effect of a constant plant-model mismatch. To that purpose, the offset-free ZMPC (denoted as OF-ZMPC) was previously developed for discrete systems in[26, 27] and for impulsive systems in [15]. This strategy aims to obtain some information about the mismatch and provide it to the ZMPC so that it corrects its prediction model and target.

To that end, the nominal model of the plant is augmented with a model that takes into account the disturbances in the form of an integrating state ($\dot{d}(t)=0$). Denote ${\tilde{x}}^{•}={\left[\begin{array}{cc} x^{•} & d^{•} \end{array}\right]}^{\prime }$ as the augmented state, where the disturbance $d\in {\mathbb{R}}^{n_{d}}$ has associated matrices $B_{d}\in {\mathbb{R}}^{n_{x}\times n_{d}}$ and $C_{d}\in {\mathbb{R}}^{n_{y}\times n_{d}}$. Then, the extended system has the form:

$$\begin{array}{cc} {\tilde{x}}^{•}(k+1)\;\;\; & ={\tilde{A}}^{d}{\tilde{x}}^{•}(k)+{\tilde{B}}_{u}^{d_{1}}u^{•}(k)+{\tilde{B}}_{r}^{d}r^{•}(k)+\tilde{E^{d}},\\ \tilde{y}(k)\;\;\; & =\tilde{C}{\tilde{x}}^{•}(k), \end{array}$$ (3)

where ${\tilde{A}}^{d}=\left[\begin{array}{cc} A^{d} & B_{d}^{d}\\ 0 & I \end{array}\right]$, ${\tilde{B}}_{u}^{d_{1}}=\left[\begin{array}{c} B_{u}^{d_{1}}\\ 0 \end{array}\right]$, ${\tilde{B}}_{r}^{d}=\left[\begin{array}{c} B_{r}^{d}\\ 0 \end{array}\right]$, ${\tilde{E}}^{d}=\left[\begin{array}{c} E^{d}\\ 0 \end{array}\right]$, $\tilde{C}=[\begin{array}{cc} C & C_{d} \end{array}]$, and $B_{d}^{d}≜e^{AT}B_{d}$.

To consider the mismatch in the optimization problem, the state and disturbance have to be estimated. Therefore, it is necessary to select matrices *B*_*d*_ and *C*_*d*_ to ensure that the augmented model is observable. This is guaranteed if and only if the augmented Kalman Observability matrix has full rank according to Proposition 1 in [15]. Then, an estimator for the state and disturbance is designed in the form:

$${\hat{\tilde{x}}}^{•}(k+1)={\tilde{A}}^{d}{\hat{\tilde{x}}}^{•}(k)+{\tilde{B}}_{u}^{d_{1}}u^{•}(k)+{\tilde{B}}_{r}^{d}r^{•}(k)+{\tilde{E}}^{d}+L(y(k)-\tilde{C}{\hat{\tilde{x}}}^{•}(k)),$$ (4)

where matrix *L* is chosen so that the estimator is stable. Here, the Kalman filter algorithm is used to obtain the estimation.

The OF-ZMPC control strategy consists of a baseline MPC and the offset-free component. The baseline formulation selected is the ZMPC developed for impulsive systems in [13]. Its main idea is to steer the system from an initial point to a target set *X*^*Tar*^ through the equilibrium points using artificial/intermediary steady state variables $(x_{a},u_{a})\in X_{s}^{•},U_{s}^{•}$. The cost function of the problem is:

$$V_{N}=V_{dyn}(x^{•};u,x_{a},u_{a})+V_{f}(X_{s}^{•Tar},U_{s}^{Tar};x_{a},u_{a}),$$ (5)

which is composed by two sections: *(i)* the dynamic cost,


$$V_{dyn}(x^{•};u,x_{a},u_{a})=\sum_{j=0}^{N-1}\|x^{•}(j)-x_{a}{\|}_{Q}^{2}+\sum_{j=0}^{N-1}\|u^{•}(j)-u_{a}{\|}_{R}^{2},$$

which steers the state to the artificial equilibrium inside $\left(X_{s}^{•},U_{s}^{•}\right)$, and *(ii)* the terminal cost,


$$V_{f}(X_{s}^{•Tar},U_{s}^{Tar};x_{a},u_{a})=P(dist_{X_{s}^{•Tar}}(x_{a})+dist_{U_{s}^{Tar}}(u_{a})),$$

which forces the artificial variables to an equilibrium that maintains the output *y*(*t*) in the target set $Y^{Tar}=CX_{s}^{•Tar}$, where *dist*_*Z*_(*x*) denotes the distance from a point *x* to set *Z*, and $X_{s}^{•Tar}$ is the maximum equilibrium set of the sampled model, such that its equilibrium trajectories remain inside *X*^*Tar*^.

Next, this baseline formulation is merged with the offset-free feature by using the augmented model as the prediction model. Given the current estimate of the augmented state ${\hat{\tilde{x}}}^{•}$, the optimization problem that solves the OF-ZMPC every time *k* is:

$$\begin{array}{cc} \min_{u,x_{a},u_{a}} & V_{N}(x^{•},X_{s}^{•Tar},U_{s}^{Tar};u,x_{a},u_{a})\\ s.t.\; & x^{•}(0)={\hat{x}}^{•}(k),\;\;d^{•}(0)={\hat{d}}^{•}(k),\\ & x^{•}(j+1)=A^{d}x^{•}(j)+B_{u}^{d_{1}}u^{•}(j)+B_{r}^{d}r^{•}(j)+B_{d}^{d}d^{•}(j)+E^{d},\\ & d^{•}(j+1)=d^{•}(j),\\ & u^{•}(j)\in U,\;x^{•}(j)\in X,\\ & x^{•}(N)=x_{a},\\ & y_{a}=Cx_{a}+C_{d}d^{•},\\ & x_{a}=A^{d}x_{a}+B_{u}^{d_{1}}u_{a}+B_{d}^{d}d^{•}+E^{d} \end{array}$$ (6)

where the first element of the solution sequence $u=\{u^{•}(0;x),\ldots ,$ $u^{•}(N-1;x)\}$ is applied to the plant every time *k*. The constraint $x^{•}(N)=x_{a}$ in (6) forces the state at the end of the horizon to reach the artificial equilibrium *x*_*a*_, and note that the plant-model mismatch is taken into account in both the prediction model and in the constraints $x_{a}=A^{d}x_{a}+B_{u}^{d_{1}}u_{a}+B_{d}^{d}d^{•}+E^{d}$ and $y_{a}=Cx_{a}+C_{d}d^{•}$ to compute the artificial variables.

The programming of the MPC control algorithm, along with the entire interface, was performed in Python 3.9.13, and the optimization package used to solve the MPC problem is Quadprog v 0.1.12. This package (*Quadratic Programming Solver*), for Python is a repository that is found for free under license (GNU *General Public License* v2.0) in the GitHub platform [28], for the programming of the dual algorithm based on the Fortran programming language and converted to C to be executed in Python.

### Ethics statement

The Ethics Committee for Animal Experimentation of the University of Antioquia grants ethical endorsement, as stated in session minutes No. 158 of April 16, 2024, for the development of the project “Evaluation of an automatic glucose regulator in a rat model with type I diabetes” where the commitment to good animal management in research activities is guaranteed.

### Animal models and housing

A control group is not included in this work. The first reason is to minimize the number of animals involved in the experiment, and the second is that the information on the glucose ranges in healthy and diabetic rats regulated by a standard bolus-basal treatment has already been documented [16, 22], so it would not add new insights to the study.

The experimental unit is a single animal. Fourteen male Wistar rats were included in this study, maintained under specific pathogen-free microbiological conditions, aged between 14 and 16 weeks old, with an average weight ranging from 300 to 500 grams. Each rat was individually housed in polycarbonate cages measuring 45 cm in length, 25 cm in width, and 20 cm in height to prevent the removal of devices. The macroenvironment conditions were temperature-controlled at 21^°^C +/- 2^°^C, controlled relative humidity of 50-65%, controlled air with 16 to 20 air changes per hour, and artificial lighting with white light on a 12/12 hours light/dark cycle, regulated by a timer. One of the rats had a health issue before starting, so data was only collected from 13 subjects. This is discussed further below.

All animals used in this study were male Wistar rats. Male rats were selected due to their increased sensitivity to the diabetogenic effects of streptozotocin (STZ), which results in more reliable and consistent induction of diabetes compared to females [29]. Prior studies have shown that male rats develop persistent hyperglycemia more readily and at lower STZ doses, reducing interindividual variability and improving the reproducibility of experimental outcomes [30]. This biological susceptibility, particularly in Wistar and Sprague-Dawley strains at younger ages, facilitates the manifestation of key diabetic symptoms such as polyuria, hyperglycemia, and weight loss, which are key indicators for confirming disease onset and progression [31].

The rats were fed Laboratory Autoclavable Rodent Diet (LabDiet^®^ 5010), which contains a minimum of 23% crude protein, 4.5% crude fat, and a maximum of 6% fiber, initially provided *ad libitum*, along with pre-sterilized water.

### Experimental procedures

When the animals left the breeding facility for experimentation, they underwent a one-week acclimatization period and became familiar with their handlers. The macroenvironmental and microenvironmental conditions were monitored daily to ensure they were ideal. The supply of food and water in adequate quantity and quality and clean, dry bedding was guaranteed. The animals were permanently monitored by trained personnel knowledgeable and experienced in the procedures to be carried out. The animals were familiarized with the trained personnel in charge of the trial, who had experience properly handling and managing the species used. The research staff had received special training for this experiment, including Working with rats in research settings, a laboratory animal care internship program, working with IACUC 1—Lab Animal Research, ethics, and handling laboratory animals.

To induce type I diabetes, the rats were fasted for six hours before being administered STZ at a dose of 60 mg/kg via intraperitoneal injection (IP) under anesthesia with isoflurane. Afterward, a 10% sucrose solution was provided in their water bottles for 24 hours, followed by regular water. Once glucose measurements exceeded 300 mg/dL, confirming type 1 diabetes in the rat, the sensor, transmitter, and insulin cannula were installed in the ventral area of each rat under anesthesia using ketamine/xylazine at 90 mg/kg and 5 mg/kg, respectively, by IP route. The sensor and cannula were secured with gauze and adhesive tape to minimize stress during handling. The total duration of the experiment was eight days from the moment the procedure was performed by injecting STZ. During the experiment, the animals were monitored three times a day. If they showed signs of hypoglycemia or alterations, they were monitored every thirty minutes.

After the procedure, the rats were returned to their individual cages for recovery. Blood samples from the saphenous vein were taken to ensure the sensor values fell within the normal accuracy range of BG of [80–180] mg/dL ± 20 mg/dL. Additionally, a manual insulin injection test was performed through the cannula to verify the pump and sensor’s proper function, with the results documented in the interface. The only insulin used was NovolinR^®^ (human recombinant DNA), a short-acting insulin delivered through the cannula in the ventral area for use with the pump.

Reaching endpoint criteria, including any signs or symptoms that led to deterioration in the animal’s welfare, was also considered. Animals showing severe pain, suffering, or distress were euthanized. Observations included changes in the skin and coat (redness, sagging, rashes, piloerection), in the eyes and mucous membranes of the mouth and conjunctiva (color and appearance), tremors, convulsions, paralysis, weight loss, increased aggression or depression, extended periods of sleep, aggressive or angry vocalization and high-pitched squealing and licking/protecting painful areas, and behaviors that indicated pain according to the Federation of European Laboratory Animal Science Associations (FELASA). At the time corresponding to the end of the experiment or reaching endpoint criteria, the rats were euthanized in a *CO*_2_ chamber with a dose of 10% to 30% of the chamber volume per minute. The autopsy was not performed until death had been confirmed.

If the research staff discovered a deceased animal, the principal investigator or designated personnel were notified immediately. The date, time, and location of discovery were recorded. The animal’s ID and cage number were noted. A gross external examination was conducted to look for signs of illness, injury, or distress (signs of infection, wounds, discharge). Necropsy Requirement: A necropsy was performed on all animals that died unexpectedly to determine the cause of death.

Fourteen rats were used for the experiment; however, one of the rats experienced health issues after the device installation procedure. The rat had a severe acute lung injury after inhalation of anesthesia to perform the procedure for installing the devices, an endpoint criterion for performing euthanasia, and resulting in data for thirteen rats where the experiment could be completed.

For the tests, after verifying the installation of the devices and the free movement of the rat in the cage, an insulin bolus was injected using the pump. First, this was done to lower the high BG levels caused by the installation procedure and the anesthesia administered, and second, to perform a parametric estimation. After the rat awoke and one hour had passed for sensor warm-up, the rat was provided with water but no food to identify only the effect of insulin on BG levels. Once the BG values stabilized within the normoglycemia range, the rat was given food at will, and the time of ingestion was recorded, though not the exact amount of carbohydrates. During the first few hours, a standard treatment was performed by administering periodic boluses to continue acquiring data that helped with parameter identification and to record the performance of the standard treatment using only the insulin pump. CGM calibrations were also performed when BG values were between 80 and 180 mg/dL.

With the data obtained on the first day, an offline parametric identification was performed, aiming to identify the critical parameters: *p*_0_ (endogenous glucose production), *p*_1_ (hepatic autoregulation), and *p*_2_ (associated with insulin sensitivity). The rest of the parameters were adjusted; however, it was assumed that the error resulting from the unknown exact amount of carbohydrates ingested and the limited quality of the data available from the subject in such a short time would contribute to model uncertainty. This uncertainty was handled by the controller through an offset-free approach.

With the initial diabetic rat model, simulations were carried out using the identified parameters to find a tuning that allowed BG regulation in the rat model. This was done over a 3-day scenario with parametric variations and different carbohydrate intake amounts throughout the simulation, as described in [14]. After determining the controller and estimator tuning parameters through simulations, real closed-loop tests of the controller began on the second day under supervision. During the first few hours, each insulin bolus calculated by the controller was manually confirmed via the interface to avoid potentially severe hypoglycemia due to model uncertainty or poor controller tuning.

Once several supervised injections were made and normoglycemia was maintained, manual confirmation was removed, and the controller switched to fully autonomous mode. Human supervision continued throughout the experiment, but the insulin amounts to be injected by the pump were no longer manually confirmed every 5 minutes. During the test, continuous monitoring was performed from the interface to assess the status of the equipment (sensor, actuator) and the rat’s condition (free movement, water, and food intake).

**Fig 2:**
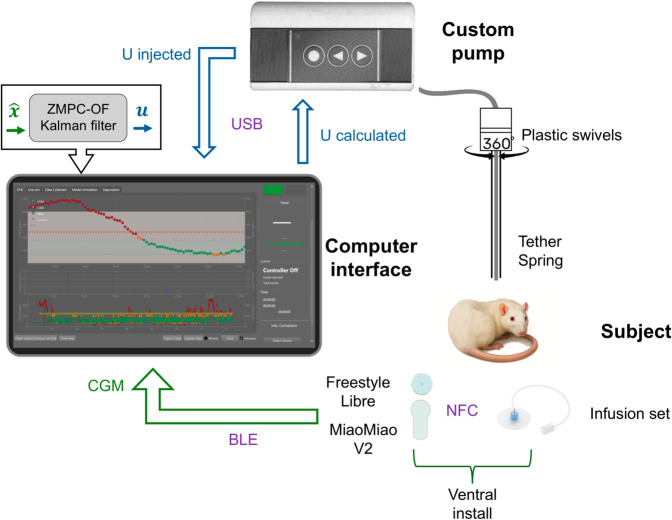
OF-ZMPC close loop test. The plastic swivels and tether spring protect and prevent the infusion set from tangling, allowing a 360^°^ rotation of the rat. The protocol between the pump and the computer interface is Universal Serial Bus (USB). The protocol between MiaoMiao V2 and the computer interface is Bluetooth Low Energy (BLE). The protocol between FreeStyle Libre and MiaoMiao V2 is near-field communication (NFC). The figure contains images that are similar but not identical to the original image and are therefore for illustrative purposes only.

The fully autonomous controller tests were carried out over three days, with water and food provided ad libitum. Due to the difficulty in measuring the exact carbohydrate intake, food intake was not announced to the control system. In Fig 2, the closed-loop control scheme of the i-AiDS was shown. Every 5 minutes, the CGM data from the MiaomiaoV2 transmitter, obtained from the Freestyle Libre sensor installed on the rat’s ventral side via Bluetooth Low Energy protocol, was recorded on a computer interface.

Using this information, state estimation was performed through a Kalman filter, and the insulin dose to be injected was calculated by the OF-ZMPC controller. The calculated units were sent via RS232 to the customized pump, which injected the insulin and returned the exact amount injected (an alarm was triggered in case of obstruction). Additionally, the interface calculated a 5-minute projection using a mathematical model of a T1DM patient with the estimated parameters. All mentioned data were stored in a database and visualized through graphs and tables in the interface.

The devices used for sensor and cannula installation allowed the rat to move freely. However, the tape, the sensor’s weight, and the connection to the cannula were factors that increased stress levels, particularly during the first few days as the rat got accustomed to them. For this reason, constant checks were performed on the rat’s behavior, carbohydrate intake, and weight at the beginning and end of the experiment. Additionally, sterilized plastic and cardboard items were provided to help reduce stress levels. Handling was minimized, and blood samples were only taken when necessary to intervene in cases of potential hypoglycemia.

### Data analysis

The number of animals used in this study (*n* = 14) was determined using a precision-based sample size calculation [32]. Based on previous studies employing closed-loop insulin delivery in STZ-induced diabetic rats [16, 22]. It is expected that the mean blood glucose level will be within the 90-130 mg/dL target range, with a standard deviation (SD) of approximately 11.5 mg/dL. The sample size was calculated to estimate the mean glucose with a margin of error of ±6 mg/dL at a 95% confidence level using Eq 7.

$$n={\left(\frac{Z_{\alpha /2}\cdot \sigma }{d}\right)}^{2}$$ (7)

Where:

- $Z_{\alpha /2}=1.96$ (for 95% confidence)
- σ=11.45 mg/dL (expected SD)
- *d* = 6 mg/dL (desired precision)

$$n={\left(\frac{1.96\cdot 11.45}{6}\right)}^{2}\approx 14$$ (8)

From Eq 8, fourteen animals were therefore included to ensure accurate estimation of glycemic control outcomes, while also accounting for potential technical failures or data exclusions. During the first day of the in-vivo test, insulin is administered at a rate of 0.9 U/Kg to lower BG levels, which initially average >300 mg/dL after the installation procedure. This also helps gather the necessary data to perform a partial parameter estimation after filtering the CGM signal. The estimated values for *p*_0_, *p*_1_, *p*_2_, and *p*_4_ are reported, along with the average values across the model of the thirteen rats, and the upper and lower bounds of the search range are also provided. Simulations are conducted for each model to determine the controller and estimator tuning, reporting the control and prediction horizons, as well as common parameters among the models.

On the second day of the test, adjustments are made to the estimated parameters to reduce the error in the 5-minute projections of the model compared to the CGM signal. The Median Absolute Relative Difference (MedARD) and its confidence interval (CI) are used to compare the projections with the actual values. During the third and fifth days, the controller tests are conducted in fully automatic mode. The average BG value, along with its SD and coefficient of variation (CV), is reported. Additionally, the percentage of time spent in the [80-180] mg/dL range is presented, as well as the instances of hypoglycemia and hyperglycemia, including their SD. Since carbohydrate intake is *ad libitum*, the regulation results for each rat over the three days are also reported. A box plot of the insulin used per day and the mean values with CI is used to show the consistency of the controller in the regulations, and Pearson’s correlation coefficient was used to assess linear relationships between continuous variables, including body weight, insulin delivery, glycemic variability, and weight change. A p−value <0.05 was considered statistically significant.

## Results

### Parameter estimator

For parametric estimation, an insulin bolus between 0.6 and 1.5 U is injected, and the time window is recorded during which normoglycemia is reached, with 37 to 80 data points corresponding to 3 to 6 hours without carbohydrate intake. These data segments are separated and analyzed individually. Before estimation, data cleaning is performed.

**Fig 3:**
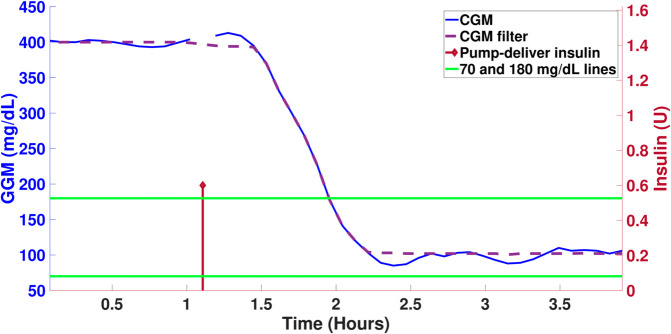
Filter signal of CGM preprocess. Left y-axis, blood glucose values reported by the sensor. Right y-axis, insulin units injected by the pump.

The first step is to address missing data, which may result from connection issues with the interface or asynchronous recording of the injected insulin bolus. This is handled using linear interpolation. Then, outliers are removed, followed by smoothing using a moving median with a smoothing factor of 0.25. The results of the smoothing process are presented in the Fig 3.

After cleaning the data, the parametric estimation process begins. Initially, since no baseline data is available, a surrogate optimization is used to perform an initial exploration to find global minima. The initial seed for this optimization comes from parameters previously identified in [24] for human patients. Parameter limits are kept open to allow the algorithm to approximate a solution within 200 iterations.

Following this, the parameters found in the first iteration are used in a nonlinear least squares method with a trust-region algorithm, applying a tolerance parameter of 0.001, a function tolerance of 0.001, and a maximum of 40 iterations. The search ranges for the parameters are limited as follows: *p*_0_ [0-20], *p*_1_ [0-0.1], *p*_2_ [0-2000], *p*_4_ [0-100]. This is based on the understanding that the parameters affecting data behavior can only be explained through endogenous glucose production, insulin sensitivity, and hepatic autoregulation. Parameters *p*_3_ and *p*_5_ show negligible variation during iterations, so they are kept constant at 9.5 and 20.45, respectively. The values of the parameters for each rat and the average of the estimated models are shown in Table 2. The table shows that the parameter with the most significant relative variation is *p*_1_, followed by *p*_0_. However, if the data are normalized, the most significant variation in the parameters occurs in *p*_0_, which represents the endogenous production of glucose, followed by *p*_2_, which represents insulin sensitivity.

**Table 2:** Parameters of the model identified for each rat.

	p0	p1	p2	p4
**DR1**	2.48	0.01	368.12	4.95
**DR2**	0.98	0.01	425.84	2.47
**DR3**	11.14	0.02	1439.53	6.23
**DR4**	7.11	0.02	543.48	3.61
**DR5**	9.21	0.03	802.50	4.29
**DR6**	7.84	0.02	732.32	8.87
**DR7**	1.73	0.00	1080.85	6.75
**DR8**	2.05	0.01	318.11	6.09
**DR9**	6.39	0.02	390.04	10.49
**DR10**	8.64	0.02	810.89	6.06
**DR11**	0.85	0.00	473.25	5.12
**DR12**	6.94	0.01	247.05	7.73
**DR13**	13.50	0.04	328.73	9.25
**CV**	0.65	0.67	0.55	0.53
**AVG**	**6.07±4.12**	**0.01±0.01**	**612.36±349.06**	**6.30±2.32**

Average values are reported as means ± SD, DR: diabetic rat; AVG: average; SD: standard deviation; CV: coefficient of variation.

With the identified parameters, the controller is tuned using a 72-hour simulation scenario featuring sporadic carbohydrate intakes between [1-5] g with intervals of [2-4] hours, starting from an initial hyperglycemic condition of 400 mg/dL. Multiple iterations are performed to find the tuning values, resulting in a prediction horizon *H*_*p*_ = 100 and a control horizon *H*_*u*_ = 30, The sampling time is maintained at 5 minutes, and restrictions are set for insulin amounts between [0-1] U, with the target BG range fixed between [90-130] mg/dL. In the estimator, the weight matrix is assigned values that reflect higher confidence in the measurements related to the model and a significant weight in the difference between the model and the actual plant *d*.

### Controller performance

The following results were obtained from testing the OF-ZMPC controller in 13 *in-vivo* experimental units as follows: After the first two days of parametric estimation and supervised controller adjustment, the 72-hour test began on the third day with the controller in automatic mode. Carbohydrate intake was unannounced, and water and food were provided *ad libitum*.

The system successfully recorded CGM data and calculated 864 insulin doses continuously throughout the 72-hour test for each subject. There were no obstruction events reported in the cannula, and the sensor and cannula installations were maintained throughout the experiment with periodic checks on the adhesive and adding extra layers if needed. On average, there was a 7-second interval from data acquisition to processing, controller calculation, and insulin injection, considering that the pump injects at a rate of 60 units per minute.

**Fig 4:**
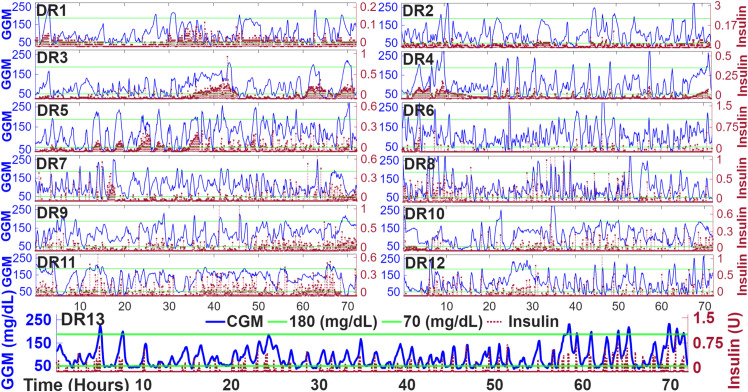
The result of fully automatic control in the thirteen rats. The green lines indicate the range 70 mg/dL≤BG≤180 mg/dL. The left y-axis shows the CGM values recorded by the interface, while the right y-axis shows the units of insulin calculated and injected by the pump. DR: diabetic rat.

In Fig 4, the results of autonomous BG regulation for each rat over three consecutive days are displayed. Peak average values of hyperglycemia are reported as 250±32 mg/dL, with a maximum of 320 mg/dL in DR6. Similarly, average peak values of hypoglycemia are 53±3 mg/dL, with a minimum of 48 mg/dL in diabetic rat (DR) 11. The average amount of insulin recorded over the three days is 31.75±11.97 U, with a maximum of 50.16 U in DR3 and a minimum of 12.64 U in DR2.

Table 3 summarizes key metrics for each rat during the 3-day experiment, including glycemic control parameters, insulin delivery, and body weight measurements. Most animals maintained a high percentage of time within the target glucose range (70–180 mg/dL), with values ranging from 76.39% to 91.55%. Instances of hypoglycemia (IG< 70 mg/dL) were observed in several animals; however, most events were mild and did not require intervention. No hyperglycemic events (IG> 180 mg/dL) were recorded. The mean insulin dose varied among individuals, with values ranging from 12.64 to 50.16 units during the study period.

**Table 3:** Glycemic control, insulin delivery, and body weight data per rat.

Rat	DR1	DR2	DR3	DR4	DR5	DR6	DR7	DR8	DR9	DR10	DR11	DR12	DR13
IG Mean (mg/dL)	113.45	110.97	100.18	110.26	126.08	117.30	117.18	110.94	128.75	119.74	137.66	114.50	99.61
SD (mg/dL)	39.52	38.17	34.49	43.38	42.79	39.16	33.51	42.70	33.13	38.53	35.32	40.30	32.98
CV (%)	34.84	34.39	34.43	39.34	33.94	33.38	28.60	38.49	25.74	32.18	25.65	35.20	33.10
PT (%) < 70 (mg/dL)	6.13	9.72	18.52	15.63	10.07	7.87	4.40	12.27	3.36	10.42	5.79	9.14	17.13
PT (%) 70–180 (mg/dL)	87.73	84.72	77.78	76.39	78.47	84.95	91.55	78.59	90.39	85.19	87.27	81.71	79.86
PT (%) > 180 (mg/dL)	6.13	5.56	3.70	7.99	11.46	7.18	4.05	9.14	6.25	4.40	6.94	9.14	3.01
Hypo	0	1	3	5	0	0	0	1	0	2	1	0	0
Mean insulin (U)	15.85	12.64	50.16	21.46	31.24	23.70	25.06	49.75	41.19	23.79	40.15	34.28	43.43
Max insulin (U)	0.11	0.13	0.86	0.19	0.32	1.50	0.77	1.26	1.50	0.77	0.65	1.50	1.01
Initial weight (g)	402.6	402.3	373.9	437.0	397.0	454.7	380.5	453.8	473.3	457.0	484.0	392.4	387.1
Final weight (g)	397.2	383.5	386.0	409.0	355.6	451.1	415.3	390.0	464.7	442.0	465.9	404.3	434.0

IG: interstitial glycemia concentration; Hypo: severe hypoglycemic events (IG< 54 mg/dL); SD: standard deviation; PT: Percentage in time; CV: coefficient of variation.

**Fig 5:**
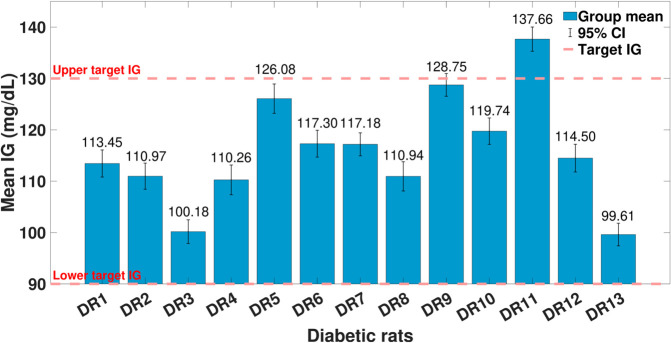
Rat-wise mean IG with 95% CI. Upper and lower targets are the controller objective range [90 - 130] (mg/dL); IG: interstitial glycemia; DR: diabetic rat; CI: confidence intervals.

The rat-wise mean IG levels with their corresponding 95% CI are shown in Fig 5. The control target range was defined as 90-130 mg/dL. Most diabetic rats maintained mean IG levels within this range. Specifically, DR5, DR6, DR7, DR9, and DR10 exhibited tightly controlled glucose values. DR3, DR4, and DR8 also remained within the target, with slightly wider CI. DR11 was the only rat that exceeded the upper target limit, with a mean IG of 137.66 mg/dL and a CI clearly outside the acceptable range. Conversely, DR13 had the lowest IG (99.61 mg/dL), still within the lower threshold. Overall, 12 of 13 rats achieved target glucose control.

The performance statistics of the controller are summarized in Table 4. The average percentage of total time in normoglycemia is 83.4±5%. The mean BG is 115.9±10.6 mg/dL, with a coefficient of variation of 33±4.2% and a *p*-value >0.05 using the Shapiro-Wilk test. No cases of severe hyperglycemia or hypoglycemia were reported, but there were 3.62±1.8 cases of hypoglycemia between [54-70] mg/dL.

**Table 4:** Statistics of the average performance of the controller in the thirteen rats.

IG Mean (mg/dL)	115.89±10.65	SD (mg/dL)	38.00±3.78	CV (%)	33.02±4.19
IG (mg/dL)	IG < 54	IG 54–70	IG 70–180	IG 180-250	IG > 250
PT (%)	0.23±0.95	10.03±4.78	83.43±5.00	6.53±2.47	0±0.61
Events	0±1.25	3.62±1.80	None	0 ± 1	0 ± 0

Values are reported as means ± SD, IG: interstitial glycemia concentration; None: in normoglycemic range there is none event; SD: standard deviation; PT: Percentage in time; CV: coefficient of variation.

When broken down by day, the average BG and insulin consumption are as follows:

Day 1: Average BG = 110.26±37.13 mg/dL with an average insulin consumption of 9.3 U.

Day 2: Average BG = 118.55±37.19 mg/dL with an average insulin consumption of 10.8 U.

Day 3: Average BG = 113.79±36.42 mg/dL with an average insulin consumption of 11.7 U.

**Fig 6:**
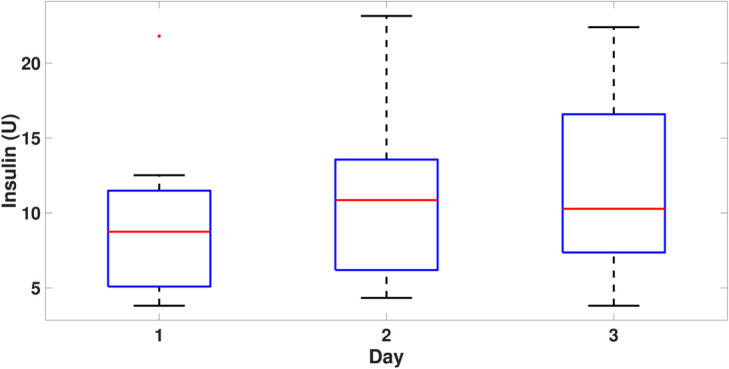
Box plot of the average insulin of the thirteen rats per day.

Fig 6 illustrates the similarity between the median insulin doses administered each day. There is also a similarity between the first and third interquartile ranges. For values beyond the 75th percentile, a maximum of 21.8 U of insulin was injected on the first day for one of the rats, which is lower than the maximum values on other days and does not represent an outlier. Despite showing positive skewness for Day 2 and negative skewness for Day 3, the insulin amounts are comparable in regulating BG across the three days for the thirteen rats.

In the tests, each CGM data point that enters the interface is used to calculate a 5-minute projection based on the implemented mathematical model. This projection helps visualize the difference between the model and the actual system (plant), and it is also estimated and sent to the controller to compensate for this difference.

**Fig 7:**
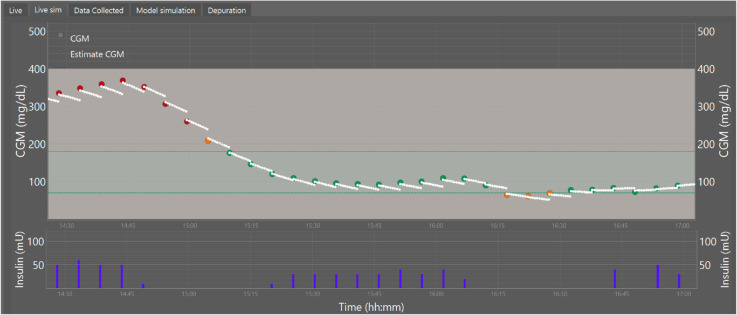
Data received from the CGM and projection to 5 minutes using the plant model. Dots in color are CGM values in ranges (Green normoglycemic in ranges 70 mg/dL ≤CGM ≤180 mg/dL), (Yellow hypo and hyperglycemic in ranges 50 < CGM and 220 > CGM) and (Red severe hypo and hyperglycemic in ranges 50 > CGM > 220). white dots are the projection using estimated parameters on the T1DM model. Bars in blue indicate the insulin that was injected.

In Fig 7, a 3-hour window shows how the projection maintains the trend of the actual data, but due to issues in parameter identification, a difference is observed. The MedARD can be used to indicate the average difference between the sensor data and the 5-minute projection calculated by the model. Since the difference between plant data and model projections does not follow a normal distribution, with a *p*-value < 0.001 using the Kolmogorov-Smirnov test for a single sample, the MedARD value of the model predictions versus the plant is 24.66% with a 95% CI of [22.47% - 26.36%] using a non-parametric technique that does not assume normality (bootstrapping) with 5000 samples.

### Rats clinical assessment

After anesthesia, rats recovered thirty minutes after the device installation procedure. They also show signs of stress due to the sensor and cannula installed on their skin, which typically results in hyperglycemia, often exceeding 300 mg/dL. This is managed by administering insulin at a rate of 0.9 U/Kg.

The rats’ weights were registered before and at the end of the experiment. Table 3 reports the weight changes for each test rat and the average, showing an overall weight loss of 4.3% from one day before STZ injection to the endpoint for each rat. In some cases, such as DR13, there is an increase in body weight of 10.8%, while in the worst case, DR8, there is a loss of weight of 14%. The mean value and standard deviation for the initial and final weights are 434±36.7g and 415.3±32.7g, respectively.

To assess whether individual differences in body weight influenced the system’s performance, we conducted a correlation analysis between initial body weight and total insulin delivered during the study period. No significant correlation was found (Pearson’s r = 0.086, p = 0.78). Similarly, changes in body weight over the three days were not significantly correlated with insulin delivery (r = 0.020, p = 0.948), indicating no significant correlation.

In the initial hours post-device implantation, some rats exhibited transient signs of stress, such as circling behavior or slow and uncoordinated movement. These behaviors subsided over time, and by the second day, animals resumed their regular circadian activity, showing reduced movement during the day and increased activity at night. Food and water intake were consistent, and no episodes of vomiting or diarrhea were observed. During episodes of hyper- and hypoglycemia, behavioral monitoring continued. No abnormal behaviors were recorded except in one rat that displayed shallow breathing and reduced mobility following anesthesia. This rat was isolated, and devices were removed. The animal was subsequently euthanized based on humane endpoint criteria. Necropsy revealed an acute pulmonary injury.

At certain times of the day and during their more active period in the dark cycle, the rats attempt to bite the devices and adhesives. Therefore, frequent inspections of the rat and the devices are performed, with additional tape applied as needed to secure the installation. This stress focus causes hyperglycemia spikes observed throughout the data in Fig 4, so not all events are due to carbohydrate intake. It is also possible that in some cases, the combination of carbohydrate intake and stress from the installation contributes to hyperglycemic events.

## Discussion

For parametric estimation, multiple signals per rat were sometimes processed due to the data quality obtained. After a 5-hour fast, food is provided *ad libitum*, which complicates the identification process due to the inability to quantify the amount of carbohydrates consumed precisely. Therefore, the parameters associated with carbohydrate intake, *p*_3_, and the maximum glucose appearance time, *p*_5_, were adjusted so that the model simulation exhibited behavior similar to that observed with the rat data and were kept constant, leaving the controller to handle this identification issue. In Table 1, the parameter *p*_2_ indicates high insulin sensitivity and generally low endogenous glucose production, which explains the amounts of insulin recorded daily. The final ranges used to restrict the search for each parameter were defined after using the surrogate search algorithm and observing general convergence within these ranges, thereby limiting search areas and possible mathematical solutions that deviate from realistic coherence.

The identified parameters can be used as a basis for simulating rats with T1DM; however, it is important to highlight the lack of information these models have regarding the response to carbohydrate intake. For this work, this deficiency in identification serves as evidence of the robustness of the controller in handling issues with the mathematical model used in the MPC, which is mitigated by the OF-ZMPC approach. Nevertheless, the model could be improved by incorporating precise information about carbohydrate intake and its effects on BG. The tuning values for the controller and estimator found in the simulation allow for regulating virtual patients without causing a computational burden on the interface or increasing the time from data acquisition to insulin injection in each iteration.

The simulations conducted with the estimated model parameters obtain tuning values and matrices for the controller. On the second day, with manual supervision, the controller is tested, and adjustments are made to the values before transitioning to full autonomy. These adjustments are also applied to the model parameters if a significant difference is observed between the 5-minute projection and the data received from the CGM. Adjustments were made to parameters *p*_0_ and *p*_2_ to improve the projection and enhance the controller’s response to BG increases.

During the controller tests, the ability to regulate IG in the thirteen rats is evident, despite the known errors in the model used by the control strategy for insulin calculation. The OF-ZMPC controller achieves an average percentage of time in range (TIR) of 83.4%±5% due to the integration of the difference between the plant and the model, *d* that has been shown to provide a reasonably accurate MedARD with a small CI that proves the discrepancy of the model in the controller and the patient during the experiment. This allows the cost function to provide a value adjusted for this discrepancy. The controller also demonstrates its ability to respond to unannounced carbohydrate intake and other disturbances, such as stress, which can cause an increase in IG levels. Figure 6 demonstrates the consistency of the controller across the experiment’s conditions. This responsiveness helps manage hyperglycemic events, with a peak reaching 320 mg/dL in DR6, but regulated to near target levels within approximately 50 minutes. For hypoglycemic events, maintaining a minimum target zone of 90 mg/dL helps to compensate for response to disturbance events without frequently causing severe hypoglycemia.

The proposed OF-ZMPC controller demonstrated a mean IG range of 99.6 to 137.7 mg/dL, with an SD range of 33–43 mg/dL, maintained over a range 77–92% TIR across animals, slight weight loss or stabilization and insulin dose (mean per rat) that varies widely from 12.6 to 50.2 U over 72 hours. Compared to other published systems, such as neural network-based MPC controllers in diabetic rats [18], which often maintained higher mean glucose levels 140–180 mg/dL with a TIR greater than 75% and the bihormonal artificial pancreas [17], with mean of 145–150 mg/dL and TIR 72–80%, our system provided tighter and more stable glycemic regulation. Furthermore, despite being monohormonal, our results compare favorably to bihormonal systems that incorporate glucagon to counter hypoglycemia.

Although we recognize that there are differences between the animal and human models, in addition to the variability in weight, sample size, and inclusion of genders, the comparison between the results obtained in this work and those presented in [33], can provide a first insight into the validation of OF-ZMPC for future clinical trials in humans. The summary of the comparison is shown in Table 5. The performance of OF-ZMPC in diabetic rats demonstrates better glycemic control than that reported in human clinical trials using the automatic regulation of glucose (ARG) algorithm. Our work presents a significantly lower mean GI and a substantially higher TIR. We also note that after normalizing the daily insulin dose per kilogram of body weight, the comparative results are as follows: Rats≈25.6U/kg/day, Humans≈0.65U/kg/day, Rats received a significantly higher insulin dose in relative terms (per kg of body weight), which is expected due to differences in basal metabolism and insulin clearance rates between species. Additionally, the STZ-induced diabetes model in rats requires more insulin to maintain normoglycemia.

**Table 5:** Comparison between OF-ZMPC performance in diabetic rats and ARG-based control in humans [33].

Metric	Rat Study (n=13)	Human Study (n=5)
Control Strategy	OF-ZMPC	ARG-based
Mean IG (mg/dL)	115.9±10.7	186.2±24.7
PT 70–180 (mg/dL)	82.5±4.4%	50.9±14.4%
PT < 70 (mg/dL)	9.9±4.4%	0.9±1.4%
PT > 180 (mg/dL)	7.6±3.1%	48.0±15.4%
Insulin (U/day)	10.6±5.5	48.5±9.7
Weight (kg)	0.42±0.034	72±9.3
Gender	Male	both

Values are reported as means ± SD; IG: interstitial glycemia concentration; SD: standard deviation; PT: Percentage in time; automatic regulation of glucose (ARG).

The analysis of rat-wise IG levels revealed that most diabetic rats maintained mean IG within the predefined therapeutic window, demonstrating effective glycemic regulation under the tested intervention. Only one rat (DR11) exhibited a significantly elevated mean IG above 130 mg/dL with a non-overlapping 95% CI, suggesting individual variability in treatment efficacy or a potential outlier. In contrast, the remaining animals sustained glucose levels either well within or close to the target limits, underscoring the reproducibility and reliability of the OF-ZMPC. The consistently narrow CI across most animals further reinforce the statistical robustness of these findings and highlight the promise of this approach for advancing i-AiDS.

A critical challenge encountered during full-automatic mode was the difficulty in monitoring food intake. With food provided *ad libitum* and rats often chewing on pellets or bedding, accurately identifying the timing and amount of food consumed was not feasible. Despite this limitation, the controller effectively regulated blood glucose levels under these unconstrained conditions, achieving outcomes comparable to studies that used movement restrictions and controlled feeding. While such restrictions can enhance experimental control, they limit ethical validity. The ability of the OF-ZMPC to function under more realistic conditions enhances its translational relevance and practical applicability.

The chosen sample size permitted a reliable estimation of system performance across individual glycemic profiles and enabled an exploratory assessment of interindividual differences. We examined whether body weight influenced insulin dosing or glycemic regulation and found no significant correlations between weight and either insulin delivery or changes in body mass. These findings support the robustness of the system across a moderate weight range. However, the relatively short study duration and limited physiological diversity may have constrained our ability to detect subtler or longer-term effects. To address this, future research should incorporate a larger and more diverse cohort of animals, monitored over extended periods, to determine whether body weight or other covariates influence system performance under varying physiological conditions.

One notable limitation of this study is the exclusive use of male rats. This choice was driven by the greater susceptibility of males to STZ and the more consistent onset of diabetes symptoms, as reported in previous studies [29–31]. However, T1DM affects individuals of all genders, and biological differences between males and females, such as in insulin sensitivity and glucose metabolism, can affect treatment outcomes [34, 35]. To ensure broader translational relevance and assess the generalizability of the OF-ZMPC, future studies should include both genders and evaluate system performance across sex-related physiological differences.

Although behavioral data were not collected using automated monitoring systems, continuous direct observation provided valuable insights into the animals’ general welfare and clinical condition. Notably, glycemic fluctuations did not lead to overt behavioral abnormalities in most animals, indicating effective physiological adaptation to closed-loop control. The only exception was a rat that developed respiratory distress unrelated to glucose control, which was appropriately managed according to humane endpoint criteria and confirmed via necropsy.

While the infusion system allowed for full mobility within the cage, the physical presence of the device introduced stress during initial adaptation, and as skin irritation developed from the adhesive over time. This necessitated limiting the experiment to five days to prevent dermal injury and cumulative distress. The development of improved attachment methods that minimize discomfort would enable longer experimental durations. Extending the trial to the full 14-day sensor lifespan would provide a more comprehensive evaluation of i-AiDS performance in prolonged scenarios, allowing for an assessment of long-term controller stability and adaptability.
